# Sexual abstinence behavior among never-married youths in a generalized HIV epidemic country: evidence from the 2005 Côte d'Ivoire AIDS indicator survey

**DOI:** 10.1186/1471-2458-8-408

**Published:** 2008-12-16

**Authors:** Alain K Koffi, Kazuo Kawahara

**Affiliations:** 1 Department of Health Policy Science, Tokyo Medical and Dental University, Graduate School of Medical and Dental Science, Tokyo, Japan

## Abstract

**Background:**

Sexual abstinence is the best available option for preventing both pregnancy and sexually transmitted infections, including HIV/AIDS. Identifying the factors associated with sexual abstinence among youths would have meaningful implications in a generalized HIV epidemic country such as the Côte d'Ivoire. Thus, we explored sexual abstinence behavior among never-married individuals aged 15 to 24 in Côte d'Ivoire and assessed factors that predict sexual abstinence.

**Methods:**

We obtained data from the nationally representative and population-based 2005 Côte d'Ivoire AIDS Indicator Survey, conducted from September 2004 to October 2005. Our sample included 3041 never-married people aged 15 to 24. Of these, 990 reported never having sexual intercourse (primary abstinence) and 137 reported sexual experience but not in the 12 months prior to the survey (secondary abstinence). In all, 1127 youths reported sexual abstinence practice.

**Results:**

Of the 3041 never-married youths, 54.4% were male and 45.6% were female. About 33.0%, 6.7%, and 37.1% of them were practicing primary, secondary, and sexual abstinence behavior, respectively. Females of higher education level were significantly 11.14 times as likely as those of no education to practice either primary or secondary abstinence. Males who were animists, had no religion, or were practicing religions other than Christianity or Muslim were significantly less likely than other male youths to practice sexual abstinence (OR = 0.53, 95% CI = 0.30–0.95). Living in the north-west region of the country significantly decreased the odds of sexual abstinence among female youths. Similarly, female youths living in rural areas were significantly 0.42 times as likely as those in the urban zones to practice sexual abstinence.

**Conclusion:**

HIV/AIDS prevention program components could include media campaigns, educational intervention improvement, as well as promoting policies that shape female youth livelihoods. Likewise, youth involvement in initiatives to design appropriate messages, and activities to promote positive behaviors or to change negative perceptions could impact on youths' decision to exert abstinence behavior.

## Background

In Côte d'Ivoire, the HIV epidemic is generalized. The country has the highest HIV prevalence in West Africa, with an estimated stable adult population prevalence of 7.1% [1]. In the country, the disease is mainly spread through heterosexual sex and now ranks second among the leading causes of death after malaria. The 2005 Côte d'Ivoire AIDS Indicator Survey (2005 AIS-Côte d'Ivoire) also revealed that premarital sexual intercourse is prevalent among youth in Côte d'Ivoire [2]. About 58% of female youths and 56% of male youths had premarital sex during the 12 months prior to the survey thereby increasing the risks associated with youth sexual activity such as sexually transmitted infections (including HIV/AIDS) and adolescent pregnancy [3-5]. Hence, the HIV prevalence rate is high among the youngest generation with females experiencing greater risks than males and rises with age reaching a peak of 10.4% for those ages 30 to 34 [2]. Moreover, because AIDS has a long latency period from infection-the average time between HIV infection and AIDS diagnosis is 7 to 9.8 years [6]-we assume people in this age group with AIDS were infected by HIV in adolescence (e.g., between the ages 15–24).

Understandably, efforts have been made to curb the spread of the infection. HIV/AIDS prevention programs promote sexual abstinence and delayed initiation of sex among never-married youth [7,8].

Unfortunately, sexual abstinence has not been clearly defined or fully examined in the literature [9,10]. Further, there is no consensus about whether sexual abstinence is a health protective behavior or something more inclusive [11]. Some researchers take a public health approach, defining abstinence as refraining from specific types of sexual contact [10]. Others are more inclusive, incorporating attitudes, moral and religious beliefs, and lifestyle choices into their definition. In one program evaluation, adults defined abstinence in behavioral terms (no vaginal, anal or oral intercourse), and youth also listed use of alcohol, cigarettes, drugs and pornography as incongruent with an abstinent lifestyle [12]. Moreover, there is no consensus on the specific sexual behaviors that define abstinence. Many adolescents and young adults do not define oral sex and other non-coital behaviors as "sex" [13,14].

Finally, contextual and developmental issues influencing abstinence are poorly understood. Research shows that adolescents differ in beliefs, attitudes, and sexual behaviors by age, gender, race/ethnicity, educational status and sexual experience [15-23]. Family and peer factors are also particularly influential in predicting adolescent sexual behavior [24-26].

Studies of interventions to delay sexual onset find differences in outcomes between males and females, and between sexually experienced and inexperienced participants [27-29]. However, though documented epidemiologically, little is known about why and how these differences arise. Besides, the reasons why some adolescents abstain from sexual intercourse and others do not are still unclear. Therefore, research need to more clearly define sexual abstinence, as well as those factors that may promote or discourage these early activities.

This research sought to extend previous research by examining the theoretical and empirical evidence of the roles of specific socio-demographic, and other contextual factors on abstinence behavior among never-married individuals aged 15 to 24 in Côte d'Ivoire. Due to the paucity of information available on the sexual behavior of this group, particularly in the Sub-Saharan Africa region, findings of this research increase the understanding of youths' sexual abstinence behavior and can enhance preventative measures.

## Methods

### Data source

Data came from the nationally representative and population-based 2005 Côte d'Ivoire AIDS Indicator Survey (2005 AIS-Côte d'Ivoire) [2]. The 2005 AIS-Côte d'Ivoire was initiated by the Ministry of the Fight against HIV/AIDS, and executed by the *Institut National de la Statistique *(INS) from September 2004 to October 2005. Technical assistance was provided through the MEASURE DEMOGRAPHIC AND HEALTH SURVEY (DHS) program, a project sponsored by the United States Agency for International Development (USAID) to collect, analyze, and disseminate population and health data. The data are accessible with permission from the MEASURE DHS website.

In order to calculate key indicators related to HIV/AIDS, the survey aimed at sampling the national and general population of reproductive age. Thus, the 2005 AIS-Côte d'Ivoire sample was stratified, clustered and selected in two stages. In the first stage, 249 clusters (109 urban and 140 rural) were selected from the 1998 Population and Housing Census sample frame. In the second stage, a complete household listing was carried out in each selected cluster with equal probability. The total sample includes 4573 households, 5183 women and 4503 men aged 15–49. These individuals were allocated roughly equally across all eleven regions of the country and personal interviews were conducted for all of them.

During the interviews, individuals were asked about their knowledge/awareness, attitudes, and behaviors regarding HIV/AIDS. Households' socio-economic and demographic characteristics were also collected. And in addition, respondents were asked to provide a few drops of blood for subsequent HIV testing in the laboratory. The testing procedure is discussed in detail in the 2005 AIS Côte d'Ivoire final report [2].

### Study population

The 2005 AIS-Côte d'Ivoire data allow the use of sample weights when nationally representative estimates are desired. The application of these weights compensates for threats to external validity inherent in Ivorian youths' non- participation and non-responses.

**Figure 1:**
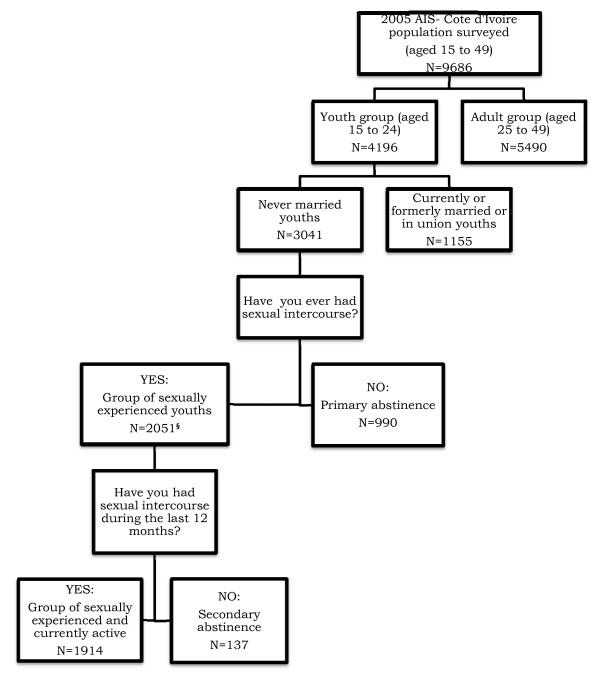
**Study sampling (weighted data)**. ^§^: Including those four female youths whose information on whether they ever had sexual intercourse was treated as missing.

Figure 1 describes our study sample, using weighted data.

This study aimed to obtain descriptive information on abstinence behavior among never- married youths. Thus, we restricted our population to never-married individuals aged 15 to 24 years old. We used two items to classify youths: whether never-married youths ever had sexual intercourse; and, among those who had sexual intercourse, whether they had sexual intercourse during the 12 months prior to the 2005 AIS Côte d'Ivoire survey.

Our weighted sample consisted of 3041 never-married people aged 15 to 24 years old. Among these youths, 990 declared they never had sexual intercourse (primary abstinence practice) and 2051 youths declared they had (sexually experienced youths), including those four female youths whose information on whether they ever had sexual intercourse was treated as missing. Among the latter, 137 young people reported that, during the previous 12 months, they did not have sexual intercourse (secondary abstinence practice). In all, 1127 never-married youths adopted the sexual abstinence behavior, either primary or secondary abstinence. The remainder (N = 1914) were classified into the group of sexually experienced and active youths.

### Measures

The outcome measures included both primary and secondary abstinence practices, and sexual abstinence behavior. Each of these variables was examined as a dichotomous (yes or no) variable. Accordingly to our typology, those who fell into the "no" category of primary abstinence included the group of sexually experienced youths, and those in the secondary abstinence group consisted of sexually experienced and active youths. Understandably, the "no" category of the sexually abstinence group also comprised sexually experienced and (currently) active youths.

The explanatory variables consisted of demographic characteristics, including age, education, religion, place of residence, geographical region of residence and regular mass media exposure (including exposure to radio, television, newspapers or magazines at least once a week). In addition, we included some psychosocial variables that have been theoretically and empirically documented to influence reproductive health behavior. Other researchers have identified psychosocial variables or ideational determinants that influence the timing of sexual debut and the circumstances surrounding sexual intercourse [30,31]. These variables included pressure to have or not have sex, perceived peer behavior, attitudes toward premarital sex, discussion about abstinence, perceived social approval of premarital abstinence, perceived self-efficacy to refuse sex under specific circumstances, and personal advocacy in favor of sexual abstinence.

In the current study, we considered some variants of these psychosocial variables, such as knowing someone who has HIV or has died of AIDS, the perceived practice of abstinence, and faithfulness among young and adult Ivoirians. The latter indicators were derived from question that assessed the following: whether most young men or women respondents know wait for sex until marriage; whether most unmarried sexually active men or women they know have only one partner; and whether most married men or women they know only have sex with their wives or husbands.

Lastly, we included questions assessing respondents' knowledge of HIV/AIDS prevention and transmission methods. For prevention methods, we included specific questions about whether it is possible to reduce the chances of getting the AIDS virus by having just one faithful uninfected sexual partner, by using condom at every sexual encounter, and by abstaining from sex. We also considered questions assessing the prevalence of common misconceptions about AIDS and HIV transmission. Respondents were asked whether they think it is possible for a healthy looking person to have the AIDS virus. They were also asked whether a person can get HIV/AIDS from mosquito bites or by sharing food with a person who has AIDS. We coded correct responses as one and constructed a HIV/AIDS related knowledge scale by adding correct responses to the questions. The scale ranged from 0 to 6, with larger values indicating the respondent had more knowledge about HIV/AIDS. The Cronbach's alpha for the scale was consistent at 0.56.

### Analysis

The descriptive statistics in this study were calculated with weighted analytic sample of 3041 never-married youths. A bivariate analysis explored the association between the dependent variables and the main explanatory variables. We then used multivariate logistic regression to analyze the independent contribution of each factor in predicting sexual abstinence behavior. Results were based on the unweighted sample for bivariate and multivariate analyses. Furthermore, because prior studies showed significant differences between males and females, we conducted the bivariate and multivariate analyses separately by gender.

## Results

The characteristics of the study sample are presented in Table 1. The 2005 AIS-Côte d'Ivoire revealed that of the 4196 youths in the survey, 72.5% had never been married. Among the latter (N = 3041), 54.4% were male and 45.6% were female. Male youths in our sample tended to be older and more educated than their female counterparts. About 33.0%, 6.7%, and 37.1% of our study sample were practicing primary, secondary, and sexual abstinence behavior, respectively. More than 80% of youths reported themselves as either Christians or Muslim. Slightly more than one-half of youths lived in urban areas, and significantly more women than men lived in these areas. Similarly, more females were living in the Abidjan-town. Data also showed that utilizing the media (radio, television and newspaper or magazine) would make it possible to reach about nine-tenths of male and female youths.

**Table 1:** Means and percentages of selected variables of never-married youths by gender

	Total N = 3041	Male N = 1653	Female N = 1388^§^	Significance of difference: (*P*-value)*
Percentages of total	100	54.4	45.6	0.000

CHARACTERISTICS				

*Dependent variables*				

Primary abstinence	32.6	31.9	33.5	ns

Secondary abstinence †	6.7	7.5	5.7	ns

Abstinence behavior	37.1	37.0	37.2	ns

*Socio-demographic characteristics*				

Mean age (yrs.)	18.80	19.25	18.28	0.000

Highest Education Level				

No education	29.3	23.2	36.7	0.000

Primary education	27.1	25.6	29.0	0.037

Secondary	39.4	45.7	31.9	0.000

Higher	4.1	5.6	2.4	0.000

Religion				

Roman Catholics	25.1	23.1	27.4	0.007

Protestants	12.4	11.5	13.5	ns

Other Christians	12.9	11.6	14.3	0.026

Muslim	30.7	32.1	29.1	ns

Others ††	18.8	21.5	15.6	0.000

Place of residence				

Urban	51.3	47.9	55.3	0.000

Rural	48.7	52.1	44.7	0.000

Region of residence				

Abidjan town	27.4	24.7	30.5	0.000

Centre	11.5	10.9	12.2	ns

Centre-East	2.2	2.3	2.2	ns

Centre-North	5.4	5.6	5.2	ns

Centre-West	10.3	11.4	9.0	0.032

North	5.2	5.5	4.8	ns

North-East	3.3	3.5	3.1	ns

North-West	2.6	3.0	2.2	ns

West	6.2	6.6	5.7	ns

South	20.4	19.8	21.0	ns

South-West	5.6	6.7	4.3	0.005

Regular exposure to mass media †††	89.9	92.4	86.9	0.000

*Ideational determinants*				

Respondent knows someone who has AIDS or has died of AIDS	12.9	14.7	10.7	0.005

Most young men wait for sex until marriage	4.6	5.0	4.0	ns

Most unmarried sexually active men have only one partner	9.1	9.7	8.5	ns

Most married men only have sex with their wives	18.6	19.8	17.1	ns

Most young women wait for sex until marriage	9.0	8.2	9.9	ns

Most unmarried sexually active women have only one partner	12.9	11.4	14.9	0.006

Most married women only have sex with their husbands	47.3	43.6	52.0	0.000

				

*HIV/AIDS-related knowledge*				

HIV/AIDS-related knowledge of preventive methods				

Abstain from sexual intercourse	79.1	78.2	80.1	ns

Limit sexual intercourse to one uninfected partner with no other partners	79.3	79.7	78.8	ns

Use of condoms	77.1	80.2	73.3	0.000

Comprehensive knowledge of HIV/AIDS transmission				

A healthy-looking person can have AIDS virus	84.0	86.5	80.9	0.000

AIDS cannot be transmitted by mosquito bites	55.1	58.0	51.6	0.001

A person cannot become infected by sharing food with a person who has AIDS	75.9	74.5	77.5	ns

HIV/AIDS knowledge and attitudes score (Mean, SD)	4.14 (1.85)	4.24 (1.79)	4.03 (1.91)	0.002

† Denominator includes the sexually experienced youths group (N = 2051); †† Including Animists, no religion or other religions; ††† Exposure to radio, television, newspapers, or magazines at least once a week. ^§: ^Including those four female youths whose information on whether they ever had sexual intercourse was treated as missing. Note: Values, unless stated, are column percentages; ns: not significant (for *P*-value greater than 0.05). *Significance levels for chi-square and *t*-tests compare males and females on each of the variables. Data derived from the 2005 AIS- Côte d'Ivoire.

More male youths significantly reported that they knew someone who had AIDS or had died of AIDS. Less than one-tenths of youths reported that most young men and women they knew were abstaining from sex until marriage. Furthermore, youths had the perception that faithfulness was more common among married women than among married men (47.3%, compared to 18.6%).

Interestingly, HIV/AIDS-related knowledge was quite high among the sample. Regarding the questions about knowledge of preventive methods (including questions about abstinence, faithfulness and condom use) 80.1% of females and 78.2% of males knew that abstinence can reduce the risk of becoming infected. About 78.8% of females and 79.7% of males knew that being faithful to one uninfected partner can reduce the risk of HIV infection, and 73.3% and 80.2%, respectively, knew that condoms use is a way to reduce the risk of HIV infection. The HIV/AIDS transmission knowledge was also high among never-married youths. Globally, the HIV/AIDS-related knowledge showed male youths had higher scores than female youths (4.2 compared to 4.0).

More than 80% of our abstinent youths had reported that they were either Christians or Muslims (Table 2). About 60% of the abstinent females lived in urban areas. A substantial proportion of youths who were exerting abstinence behavior had the perception that most of married men and women they knew had only sex with their spouses. Similarly, abstinent youths had good HIV/AIDS related knowledge. It is worth mentioning the relatively small number of cases for several variables, particularly in the secondary abstinence group of our sample, and this is a source of bias. Table 2 also explores the relationship between selected variables and abstinence behavior for both male and female youths. According to this table, all of selected variables were individually and significantly correlated with sexual abstinence practice.

**Table 2:** Associations between selected variables and youths' abstinence behavior by gender

	Male N = 1653			Female N = 1388^§^		
	Primary abstinence N = 527	Secondary abstinence N = 84†	Abstinence behavior N = 611	Primary abstinence N = 463	Secondary abstinence N = 53†	Abstinence behavior N = 516

Percentages of total	31.9	7.5	37.0	33.5	5.7	37.2

						

*Socio-demographic characteristics*						

Age groups						

15–16	47.2**	7.1	41.8**	56.4**	3.8*	51.0**

17–18	27.7**	14.3	25.9*	28.3*	13.2*	26.6

19–20	15.2**	33.3	17.6**	8.9**	41.5*	12.0**

21–22	6.5**	20.2	8.3**	1.7**	25.0*	4.3**

23–24	3.4**	24.7	6.4**	4.8**	17.3	6.0**

Highest Education Level						

No education	28.8**	19.0	27.6**	36.5**	30.2	35.9

Primary education	28.4*	15.5	26.6	27.4*	26.4	27.3

Secondary	41.0**	58.3	43.4	32.6*	43.4	33.7

Higher	1.7**	7.1	2.5**	3.5	0.0	3.1

Religion						

Roman Catholics	22.8	19.0	22.3	24.6**	25.0	24.8

Protestants	12.0	11.8	11.8	13.0	21.2	14.0

Other Christians	12.7	23.8**	14.2*	13.4	32.1**	15.1

Muslim	36.6	35.7	36.5**	32.2**	13.5*	30.4

Others ††	16.1*	9.5	15.2**	16.6	7.5	15.5

Place of residence						

Urban	44.0	56.0	45.7	60.0*	58.5	59.9**

Rural	56.0	44.0	54.3	40.0	41.5	40.1

Region of residence						

Abidjan town	20.7*	32.1	22.2	32.4	35.8*	32.8

Centre	8.5	7.1	8.3**	6.9**	18.9	8.1**

Centre-East	3.2*	4.8	3.3*	1.9	0.0	1.7

Centre-North	4.7	4.8	4.7	5.2	3.8	5.0

Centre-West	13.1	9.4	12.4	9.7	3.8	9.1

North	7.0	2.4	6.2	6.5**	1.9	6.0

North-East	4.9*	1.2	4.4	3.0	0.0	2.7

North-West	3.0	1.2	2.8	2.4	0.0	2.1

West	5.1	3.6	4.9*	5.4	0.0	5.0

South	21.8	28.6*	22.6*	22.2	24.5	22.7

South-West	8.2	7.1	8.0	4.3	7.7	4.7

Regular exposure to mass media †††	88.4**	92.9	89.2**	86.8	94.3	87.6

*Ideational determinants*	9.5**	9.0	9.6**	9.8	30.0	12.0

Respondent knows someone who has AIDS or has died of AIDS	9.2**	2.5	8.4**	7.8**	1.9	7.2**

Most young men wait for sex until marriage	12.3**	9.1*	11.9*	11.9**	2.0	10.8*

Most unmarried sexually active men have only one partner	27.3**	13.7	25.4**	34.8**	13.5*	32.1**

Most married men only have sex with their wives	15.1**	6.2	13.8**	17.1**	3.8	15.3**

Most young women wait for sex until marriage	16.6**	10.0	15.6**	22.7**	12.8	21.5**

Most unmarried sexually active women have only one partner	50.4**	35.1	48.4**	67.9**	37.8	64.4**

Most married women only have sex with their husbands						

						

*HIV/AIDS-related knowledge*						

HIV/AIDS-related knowledge of preventive methods	74.1	82.5	75.3*	76.4	84.6	77.3

Abstain from sexual intercourse	80.2	83.5	80.7	72.9**	70.6	72.6**

Limit sexual intercourse to one uninfected partner with no other partners	73.1	81.3	74.4*	61.2**	92.2	64.8**

Use of condoms						

Comprehensive knowledge of HIV/AIDS transmission	82.5**	94.6	84.4	78.1**	91.7	79.8

A healthy-looking person can have AIDS virus	52.5	60	53.7*	50.7	65.4	52.4

AIDS cannot be transmitted by mosquito bites	69.9	84.4*	71.8	73.0*	82.7*	74.2*

A person cannot become infected by sharing food with a person who has AIDS	9.5**	9.0	9.6**	9.8	30.0	12.0

**. Correlation is significant at the 0.01 level (2-tailed); *. Correlation is significant at the 0.05 level (2-tailed); † Denominator includes the sexually experienced youths group (for male, N = 1126; for female, N = 925); †† Including Animists, no religion or other religions; ††† Exposure to radio, television, newspapers, or magazines at least once a week. ^§: ^Including those four female youths whose information on whether they ever had sexual intercourse was treated as missing. Note: All the explanatory variables are categorical; Values, unless stated are column percentages. The percentage distribution is based on weighted data. Significance testing is based on the chi-square test, using unweighted data. Data derived from the 2005 AIS- Côte d'Ivoire.

In a prior version of this paper, all the selected variables were entered into logistic regression models to examine how they are associated with the likelihood of practicing primary, secondary abstinence, or adopting sexual abstinence behavior. These models were run separately by gender. The logistic regression model that relates sexual abstinence behavior to our explanatory variables fit relatively well as evidenced in the Hosmer-Lemeshow goodness-of-fit statistics, and succeeded in correctly classifying a substantial proportion of cases, by gender. Furthermore, all the explanatory variables accounted for 34.0% and 36.5% of the variance in sexual abstinence practice among male and female youths, respectively. Therefore, we chose to present only the results of this model in Table 3. The results were expressed as odds ratios (OR) with their 95% confidence intervals (CI).

**Table 3:** Multiple logistic regression on sexual abstinence behavior

	**Youth Males** **O.R (95% CI)**	**Youth Females** **O.R (95% CI)**
*Socio-demographic characteristics*		

Respondent current age	0.67 (0.62–0.73)**	0.60 (0.53–0.68)**

Highest Education Level		

No education	Ref	Ref

Primary education	0.95 (0.53–1.70)	0.68 (0.35–1.33)

Secondary	0.75 (0.41–1.39)	1.19 (0.60–2.34)

Higher	1.26 (0.41–3.83)	11.14 (2.44–50.89)**

Religion		

Roman Catholic	Ref	Ref

Protestants	1.21 (0.64–2.30)	1.03 (0.47–2.28)

Other Christian Religions	1.65 (0.92–2.97)	0.91 (0.45–1.81)

Muslim	0.87 (0.53–1.43)	1.54 (0.78–3.05)

Others †	0.53 (0.30–0.95)*	0.54 (0.21–1.41)

Place of residence		

Urban	Ref	Ref

Rural	0.71 (0.47–1.06)	0.42 (0.24–0.75)**

Region of residence		

Abidjan town	Ref	Ref

Centre	1.04 (0.49–2.19)	0.68 (0.30–1.55)

Centre-East	2.03 (0.97–4.22)	0.93 (0.37–2.31)

Centre-North	0.54 (0.19–1.51)	0.44 (0.13–1.45)

Centre-West	1.87 (0.86–4.05)	0.65 (0.24–1.81)

North	0.72 (0.21–2.50)	0.18 (0.03–1.06)

North-East	1.60 (0.71–3.61)	0.58 (0.18–1.85)

North-West	0.55 (0.19–1.55)	0.04 (0.00–0.35)**

West	0.86 (0.29–2.51)	0.57 (0.14–2.35)

South	2.02 (0.97–4.24)	1.39 (0.60–3.25)

South-West	1.26 (0.56–2.82)	0.66 (0.23–1.84)

Regular exposure to mass media ††	0.92 (0.41–2.07)	0.51 (0.22–1.17)

		

*Ideational determinants*		

Respondent knows someone who has AIDS or has died of AIDS	0.46 (0.26–0.80)**	1.66 (0.83–3.29)

Most young men wait for sex until marriage	0.80 (0.33–1.94)	1.85 (0.47–7.33)

Most unmarried sexually active men have only one partner	1.61 (0.81–3.19)	0.26 (0.07–0.98)*

Most married men only have sex with their wives	0.99 (0.59–1.67)	1.94 (0.92–4.10)

Most young women wait for sex until marriage	1.60 (0.78–3.29)	2.05 (0.75–5.60)

Most unmarried sexually active women have only one partner	0.70 (0.34–1.41)	1.21 (0.50–2.94)

Most married women only have sex with their husbands	1.23 (0.84–1.81)	0.89 (0.53–1.49)

*HIV/AIDS-related knowledge*		

HIV/AIDS-related knowledge of preventive methods		

Abstain from sexual intercourse	0.96 (0.57–1.60)	0.91 (0.45–1.83)

Limit sexual intercourse to one uninfected partner with no other partners	1.54 (0.86–2.75)	0.50 (0.26–0.98)*

Use of condoms	0.45 (0.26–0.77)**	0.71 (0.36–1.41)

Comprehensive knowledge of HIV/AIDS transmission		

A healthy-looking person can have AIDS virus	0.72 (0.43–1.20)	1.00 (0.54–1.85)

AIDS cannot be transmitted by mosquito bites	1.03 (0.66–1.59)	1.37 (0.81–2.34)

A person cannot become infected by sharing food with a person who has AIDS	1.73 (1.02–2.96)*	0.93 (0.48–1.80)

*Pseudo-R*^2^	34.0%	36.5%

*Number of cases (included in the analysis)*	820	550

*Hosmer and Lemeshow Test χ^2 ^(grouped)/p*	6.18/0.63	4.53/0.81

*% correctly classified*	76.8	80.2

** OR is significant at the 0.01 level (2-tailed). * OR is significant at the 0.05 level (2-tailed). † Including Animists, no religion or other religions; †† Exposure to radio, television, newspapers, or magazines at least once a week. Note: Reference categories, unless stated, are null categories. All significant OR are in bold. Ref: Reference category. Logistic regression analysis is based on unweighted data. Data derived from the 2005 AIS- Côte d'Ivoire.

Thus, sexual abstinence practice significantly decreased with age among never-married Ivorian youths. Females of higher education level were significantly 11.14 times as likely as those of no education to practice either primary or secondary abstinence. Males who were animists, had no religion, or were practicing religions other than Christianity or Islam were significantly less likely than other male youths to practice sexual abstinence (OR = 0.53, 95% CI = 0.30–0.95). Living in the north-west region of the country significantly decreased the odds of sexual abstinence among never-married female youths. Likewise, female youths living in rural areas, compared to females in urban areas, were significantly more reluctant to practice sexual abstinence (OR = 0.42, 95% CI = 0.24–0.75). Knowing someone who has AIDS or has died of AIDS significantly decreased the odds of sexual abstinence practice among male youths (OR = 0.46; 95% CI = 0.26–0.80). The perception of delayed premarital sex practice among young men and women was positively related to sexual abstinence among female youths. For instance, female youths who reported that most young men they knew waited for sex until marriage were 1.85 times as likely as other females to practice either primary or secondary abstinence (albeit marginally). The perceived practice of faithfulness among sexually active unmarried men increased the likelihood of sexual abstinence practice among male youths (OR = 1.61; 95% CI = 0.81–3.19), while significantly decreased that of female youths (OR = 0.26; 95% CI = 0.07–0.98). Furthermore, female youths who reported that most married sexually active men they knew only had sex with their wives were 1.94 times as likely as the other females to practice either primary or secondary abstinence. In addition, male youths who knew that condom use can prevent HIV/AIDS were significantly less likely to abhor sexual abstinence behavior (OR = 0.45, 95% CI = 0.26–0.77). Knowledge of being faithful as a way to avoid HIV/AIDS was negatively associated with sexual abstinence practice among female youths (OR = 0.50, 95% CI = 0.26–0.98). Lastly, male youths who knew that a person cannot be infected by sharing food with a person who has AIDS were significantly 1.73 times more likely to adopt primary or secondary abstinence behavior.

## Discussion

One of the principal findings from this research was that the proportion of never-married male youths was larger than the proportion of never-married female youths, which reflect the fact that females get married at earlier ages than their counterpart males in Côte d'Ivoire [2]. We found that both male and female youths similarly adopted abstinence behavior (either primary or secondary abstinence). Other studies found that abstinence was higher among female youths [31].

Another important correlate of sexual abstinence behavior is age, with older youth being significantly less likely than their younger counterparts to report sexual abstinence practice. This points to the need to target youth with either primary or secondary abstinence messages early (preferably in the early teenage years) and thereby strengthen their capability to refuse sex before sexual activity becomes widespread. As Babalola *et al*. ideally put it, critical thinking, priority setting, problem solving, sexual negotiation, and coping with life challenges constitute appropriate skills that an intervention program should help youths to develop early and apply in every day situations [32].

Interestingly, higher education was significantly and positively associated with sexual abstinence among female youths, echoing the need to improve education attainment, particularly among this group, as previously highlighted [24,33].

Female youths living in rural areas were significantly less likely than other females to abhor sexual abstinence practice. This suggests that marriages in rural areas occur at a young age and abstinence until marriage might be difficult as these areas feature communities with poor socio-economic status and less HIV/AIDS preventive behavior than urban areas. The finding also underscores the need to focus on urban youth specifically, in efforts aimed at promoting sexual abstinence. Moreover, developing holistic approaches that could shape rural female youth livelihoods is essential to empower female youth to control issues such as their sexual and reproductive health and rights.

Also notable, abstinence behavior was unevenly distributed throughout the country, with youths living in the north-west part of the country less likely to practice either primary or secondary abstinence compared to other youths. This region also has the lowest education level in the country [2], which may contribute to the high-risk sexual behavior prevalent among these inhabitants. Improving education attainment throughout the country, especially in the north-west part, is a strategy likely to foster delayed sexual debut.

The finding that male youths who were Christians or Muslims were significantly more likely than other male youths to adopt sexual behavior is quite interesting and not surprising. From the standpoint of these religions (Christianity and Islam) on sexual abstinence, sexual intercourse is meant to take place with the context of marriage; therefore, sexual abstinence is expected of unmarried people. Extensive researches have also documented the major impact of these religions on adolescents' sexual behavior [34,35]. Therefore, HIV/AIDS prevention could include religious leaders in Côte d'Ivoire to serve as reliable medium for providing comprehensive sex education not only to their congregations, but also to the larger community. Besides, these religious institutions possess some resources such as schools, hospitals, clinics and orphanages that can be tapped for HIV/AIDS prevention programs aimed at encouraging youth to postpone the onset of sexual activity at least until marriage.

The present study also showed that, contrary to youths' perceptions, a substantial proportion of them were postponing sexual debut until marriage. Since the perceived practice of abstinence among young people who female participants knew was positively associated (albeit marginally) with abstinence practice, it is wise to design appropriate messages and activities in order to correct and provide visibility to positive deviants (youths who are delaying sexual debut or practicing secondary sexual abstinence). For instance, abstinent youths should be encouraged to be proud and vocal about their behavior and to advocate in favor of sexual abstinence behavior among their peers. Another study mentioned interpersonal channels as an effective means to persuade individuals to adopt new behaviors [36]. Understandably, the use of interpersonal channels could form a substantial part of any communication campaign in Côte d'Ivoire.

However, this study has several limitations. First, the study used a cross-sectional design; thus, it is impossible to establish causality. A study using a longitudinal design is necessary to assess the significance and stability of predictors of abstinence behaviors over time. Second, the study is based on a secondary analysis with archival data, which likely reflects the perspectives and purposes of the original investigators. We were challenged to shape the data to match our research questions, which required an intensive process of understanding the data set, recoding variables, and recasting research questions to match data available. Other limitations include self-reported information, which increases the possibility of inaccuracies, particularly with regard to information about HIV/AIDS and sexual behavior. And given an increasing social pressure to delay sexual initiation and avoid pregnancy or sexually infectious diseases such as HIV/AIDS, youths may be likely to underreport their sexual activity. Therefore, caution should be used when generalizing these findings. Lastly, many studies of youth sexual behavior, including the current one, only ask about participation in sexual intercourse, leaving a gap in our knowledge regarding other sexual behaviors that young people might participate in, such as oral and anal sexual behaviors [37]. Thus, further research should attempt to measure behaviors other than sexual intercourse.

In spite of these limitations, the findings of this study are important. They contribute to the field by examining the topic of youth abstinence behavior in a sub-Saharan African context.

## Conclusion

Youth sexual abstinence is shaped by a number of interlinking forces that include individual and familial influences, as well as a network of more indirect forces, such as peers, school and community. Better identification and understanding of the interplay between these individual-level characteristics and a variety of social and cultural contexts is necessary for programmatic youth development.

Our findings demonstrate that age, education, and some religious institutions play a key role in promoting sexual abstinence. Living in rural areas decreased the likelihood of practicing sexual abstinence. The perceived practice of abstinence among young people who female participants knew was positively associated (albeit marginal) with abstinence practice.

HIV/AIDS prevention program components could include media campaigns, educational intervention improvement, as well as promoting policies that shape female youth livelihoods. Likewise, youth involvement at early ages in initiatives to design appropriate messages, and activities to promote positive behaviors or to change negative perceptions could impact on youths' decision to exert abstinence behavior. Lastly, the inclusion of Christian and Islamic leaders in HIV/AIDS work can be beneficial.

## Competing interests

The authors declare that they have no competing interests.

## Authors' contributions

AKK participated in the conception and design of the study, analyzed, and drafted the manuscript. KK participated in the conception and design of the study, and critically reviewed the manuscript. All authors gave their final approval for the manuscript submitted for publication.

## Pre-publication history

The pre-publication history for this paper can be accessed here:


